# The Effect of Low Protein Energy-Rich Diets on Plasma Hepatic Markers, Hepatic Damage, and Discrimination Reversal Learning in Young Female Chicks

**DOI:** 10.3389/fvets.2018.00107

**Published:** 2018-05-23

**Authors:** Laura Bona, Nienke van Staaveren, Bishwo Bandhu Pokharel, Marinus van Krimpen, Alexandra Harlander-Matauschek

**Affiliations:** ^1^Department of Animal Biosciences, University of Guelph, Guelph, ON, Canada; ^2^Wageningen UR Livestock Research, Wageningen, Netherlands

**Keywords:** female broiler, hepatic damage, enzymes, cognition, welfare

## Abstract

Consumption of low protein energy-rich (LPER) diets increases susceptibility to metabolic disease in mammals, such as hepatic damage, and can have an adverse effect on cognition. However, the effects of these diets on both physical and mental welfare have not been investigated in domestic meat chickens. Female chicks received a low protein energy-rich or a standard control diet from 21 to 51 days of age. The effects of these dietary manipulations on plasma hepatic markers for liver damage, liver necropsy, and learning a visual discrimination reversal task were assessed. Birds given access to LPER diets weighed less than chicks that had access to the control diets. All chicks had post-mortem signs of hepatic hemorrhage/increased liver color scores and aspartate aminotransferase (AST) levels above 230 U/L indicative of hepatic damage in birds. The LPER diet had no impact on the performance of female chicks when learning to distinguish colors in a reversal visual discrimination task. The present study suggests that liver damage does not become worse when feeding LPER or impact visual reversal learning in female meat-type chickens. However, the high incidence of liver cell damage/liver hemorrhage, and “abnormal” AST activities are of concern in female broiler chicks across both diets, and suggests that the health of modern meat-type genotypes needs to be improved.

## Introduction

People who live in “modern” industrialized societies eat what and how much they like because they have easy access to food. However, chronic consumption of large amounts of processed, energy-rich (1), and animal-based protein diets (2) have been directly linked to negative effects on human health (1, 3) in sedentary societies. For instance, protein intake greater than its safe upper limits can exceed the ability of the liver, intestine and kidneys to detoxify protein metabolites such as ammonia (4). This can lead to hyperammonemia, liver damage and fatigue (5), and includes diseases such as obesity and non-alcoholic fatty liver disease (1, 3).

Domesticated chickens, the most common domestic animals worldwide, provide an important protein source in human diets and consequently are of enormous economic value in the future (2). Modern meat-type chickens grow very fast, and gain weight at excessive rates due to genetic selection and optimized chicken diet composition (6). The level of protein and amino acids in chicken diets are determined to produce high yield, pushing muscle fibers to their limits (7), and they produce overweight birds, which are typically used as animal models for studies in obesity (8). Due to their fast growth in a short period leading to extremely high body weights, meat-type chickens experience welfare problems, including skeletal problems, leading to inactivity (9). However, society is increasingly interested in more animal –welfare friendly farmed meat (10, 11), which includes slower growth rates/lower body weights, which could be achieved by lowering the protein content of meat-type chicken diets (12) while increasing energy content. Not only could this diet composition benefit the bird by slower growth rates and less metabolic heat production, especially under hot climate (13), but could also benefit farmers in specialty/niche programs (14) by reduced feed costs, as protein is one of the costliest nutrients in poultry feed (15). Furthermore, these low protein energy-rich (LPER) diets can also benefit the environment by lowering the level of nitrogen being excreted and available for volatilization to ammonia gas (16, 17).

However, when LPER amino acid imbalanced diets are fed to meat-type chickens, final body weight, feed intake and feed conversation are negatively affected (18, 19) and make these diets economically less efficient for commercial/non-specialty/non-niche market farmers. Furthermore, feeding of low protein diets or diets with varying protein and energy ratios can have positive side effects such as reduced incidence of ascites and improved litter quality, resulting in less footpad lesions (19–21). Interestingly, a combination of reduced protein and excessive energy intake, along with inactivity can result in the development of non-alcoholic fatty liver disease, such as fatty liver hemorrhagic syndrome (FLHS) commonly seen in birds kept for egg-laying or overweight backyard chickens (22–29). However, these conditions of reduced protein and excessive energy intake can also occur in meat-type chickens and fatty liver disorders have been observed in overweight backyard chickens and broilers (27, 30–32). Moreover, several lines of research in laying hens and rats have led to the suggestion that impaired liver function can lead to an accumulation of ammonia in the blood, and an increase in plasma hepatic markers including alanine aminotransferase (ALT), aspartate aminotransferase (AST), and gamma-glutamyl transferase (GGT) (26, 33, 34). Research also indicates that there is a close association between liver enzyme activity and cognitive function in humans with non-alcoholic fatty livers (3). Such cognitive impairments due to liver disease may range from mild cognitive changes, seen in memory and attention, to overt hepatic encephalopathy (35), and have been associated with lower quality of life (36, 37). Nevertheless, there have been no investigations to our knowledge to identify whether impaired cognitive function is associated with liver disease in birds kept for egg laying or meat-type chickens.

There have been a number of studies addressing the effects of LPER diets on cognition and learning in humans and other mammals (38–40). Early research by O’Connel et al. (41) showed that protein-malnourished monkeys had impairment in information processing when performing visual discrimination tasks, and Zimmermann (42) found that animals fed low protein diets were inferior on learning reversal problems. More recently, Davidson et al. (43) and Reyes-Castro et al. (38) found that when rats were fed an energy-rich diet or protein-restricted diet, they performed more poorly on visual discrimination tasks compared to rats fed standard chow. Erhard et al. (39) also reported significantly worse performance in visual discrimination tasks when sheep were malnourished and fed a protein-restricted diet.

Despite the vast human/nutritionists knowledge about dietary requirements of protein by meat-type chickens, there is a general lack of information on how dietary protein affects liver health and cognition in these birds. We hypothesized that female chicks fed a low protein energy-rich (LPER) diet without supplemental essential amino acids will have lower final body weights, a higher risk of liver cell damage (indicated by increased plasma hepatic markers, and higher liver color and hemorrhagic scores), and will be more inferior on learning a visual discrimination reversal task compared to female chicks fed a standard control diet, as cognitive impairments have been associated with low-protein energy- rich diets in mammals. Additionally, we expected that hens with the highest activity levels of plasma hepatic markers and liver scores would be the least successful in reversal learning.

## Methods

### Ethical Statement

The experiment was approved by the Animal Care Council, University of Guelph, Guelph, ON, Canada (Animal User Protocol #3609).

### Animals, Housing, and Treatments

A total of 40 one-day old female Ross broiler chicks were housed in 4 pens (119 x 144 cm; *n* = 10/pen), under standard commercial lighting and temperature protocol. Dark brooders in the form of grey plastic containers measuring 35 x 42 x 35 cm flipped upside down with pop holes of 10 x 15 cm on the length sides were positioned in the middle of each pen during the first week of life. Pens were provided with seven automatic water nipples and one feeder (40 x 25 cm). Also, chicks were fed on paper during the first 5 days. Birds in adjacent pens were visually separated from each other by 1-meter white plastic boards. All chicks were wing tagged on day 1 to assist in identification.

**Figure 1:**
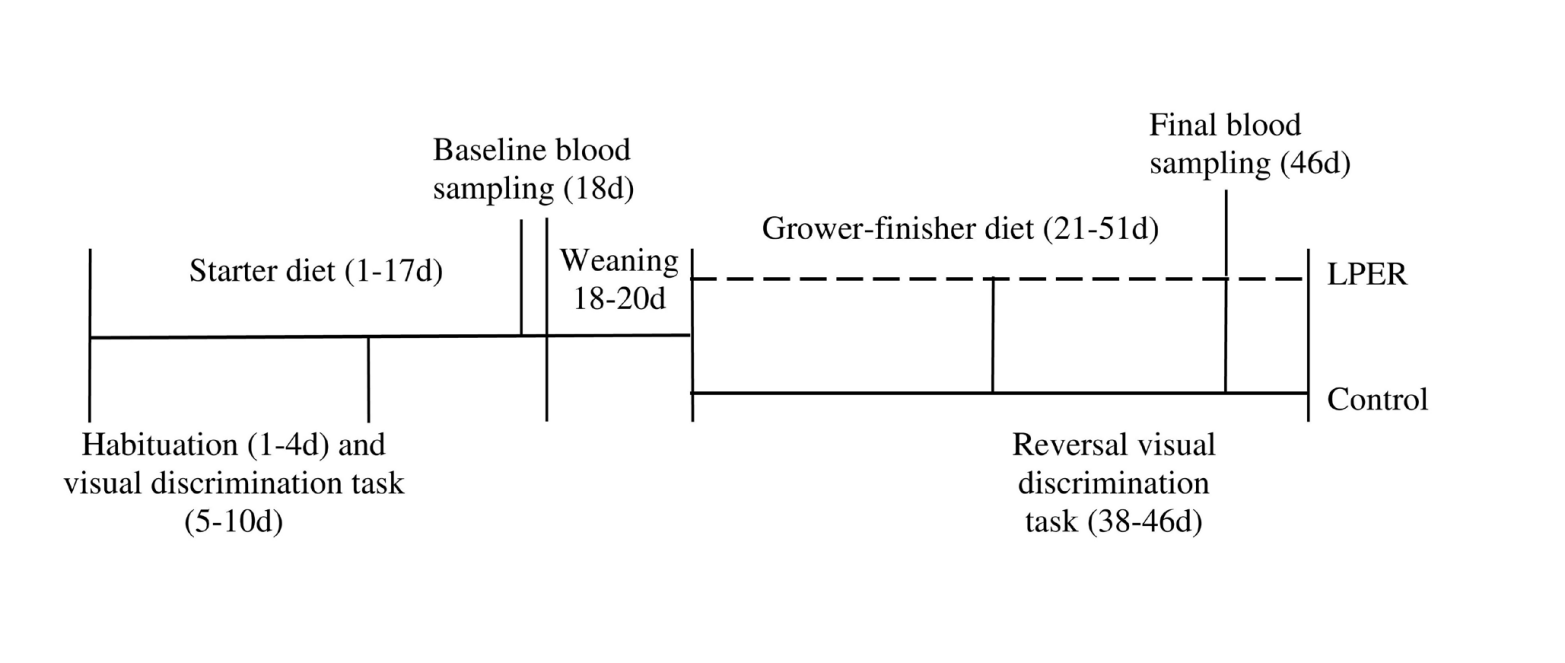
Timeline of events for entire study (1–51 d). All birds received the same commercial diet from day 1–17. Birds received a mixture of the commercial and treatment diets from days 18–20 (either control or LPER). All birds received either control (solid line) or low protein energy-rich (LPER; dashed line) diet from days 21–51. Blood sampling occurred at day 18 and at day 46. Birds underwent habituation and visual discrimination task at day 1–10, followed by reversal visual discrimination task at day 38–46.

Pens of birds were randomly assigned to the treatment diet (LPER or control) on day 1. Initially, all chicks were fed the same standard commercial diet from day 1–21. During days 18–20 all birds received a standard commercial diet followed by gradually introducing 2 parts of either the control or LPER grower-finisher diet. On day 21, pens of birds received either a control or a LPER diet (2 pens per treatment). The experimental timeline is depicted in Figure 1. Diets were corn, soybean meal, and meat meal based, formulated to meet the nutrient recommendations for Ross 308 (44), and were made at Arkell Research Station, University of Guelph, Guelph, ON, Canada. Table 1 shows the ingredient and nutrient composition of the starter and two treatment grower-finisher diets.

**Table 1:** Composition of starter, control grower-finisher, and low protein energy-rich (LPER) grower-finisher diets made in-house.

Calculated %	Starter diet (1–21 d)	Control grower-finisher (21–51 d)	LPER grower-finisher (21–51 d)
CP	21	19	17
ME, Kcal/kg	3,000	3,200	3,300
EE	10.2	12.6	13.7
Ca	0.87	0.68	0.68
AvP	0.43	0.32	0.32
Na	0.21	0.2	0.2
Lys	1.37	1.15	1.16
Met	0.5	0.48	0.48

All birds received the same starter diet from day 1–21. Pens were assigned the treatment or control diet after day 21.

### Body Weight and Feed Intake

All birds were individually weighed on day 1 and subsequently on a weekly basis to estimate average daily gain (ADG). Feed intake per pen was measured on a weekly basis to estimate average daily intake (ADI) per bird, and feed conversion ratio (FCR) was calculated for each bird.

### Plasma Hepatic Markers

On day 18 and day 46, blood samples were obtained from the wing vein of each bird between 9:00 and 9:30 am. Three mL of blood were collected using 23-gauge needles into lithium heparin vacutainer tubes and centrifuged for plasma collection (3,000 x g at 4°C for 10 min). Ammonia (NH4) was determined in deproteinized blood plasma according to the calorimetric method described in McCullough (45), as high blood NH4 concentrations may indicate reduced liver function or NH4 poisoning (46, 47). Blood plasma was acidified and deproteinized for NH4 measurments within 1 h of blood sampling, and the calorimetric reaction was performed within 12 h.

Blood samples were analyzed for elevated plasma enzyme acitivities indicating cellular damage. Analyses of enzyme profiles of alanine aminotransferase (ALT), indicating non-specific cell damage (48), aspartate aminotransferase (AST), sensitive avian indicator for liver damage and muscle damage (46) and gamma-glutamyl transferase (GGT), indicating bird liver and biliary compromises (46, 47) but are not elevated during muscle damage in pigeons (47). Plasma ALT, AST, and GGT were analyzed using the Roche Cobas C ASTL kit ID 0–494, Roche Cobas C ALTL kit ID 0–495 and Roche Cobas C GGT-2 kit version 2 (Roche Diagnostics, Indianapolis, IN, USA), respectively were investigated at the Animal Health Laboratory at the University of Guelph, Guelph, ON, Canada. As the use of test combinations improves information received with single enzyme determination, AST:ALT ratio was calculated, where it has been suggested that an AST:ALT ratio >1 indicates the presence of late-stage scarring (fibrosis) in the liver in humans (49).

## Post-Mortem Inspections

Since AST and ALT enzyme activity levels are not specific and limited to one organ (49), blood work was accompanied by post-mortem necropsy. Birds were sacrificed at 52 days of age for post-mortem (PM) inspection of the liver. Livers were weighed and hemorrhage damage and color score were determined. A JVC camera (JVC GC-PX100BU HD Everio) mounted on a tripod 44 cm above the liver was used to take pictures of all livers during PM inspection. Liver hemorrhage damage was scored as described previously by Shini (26), from 0 to 5; 0 = absolutely no hemorrhaging on the liver; 1 = less than 10 hemorrhages on the liver; 2 = more than 10 hemorrhages on the liver; 3–5 = severe hemorrhaging. Liver color was scored as described by Choi et al. (50) from 1 to 5, with 1 being a normal deep red color, and 5 being a yellow or putty-like colored liver.

## Test Apparatus

**Figure 2:**
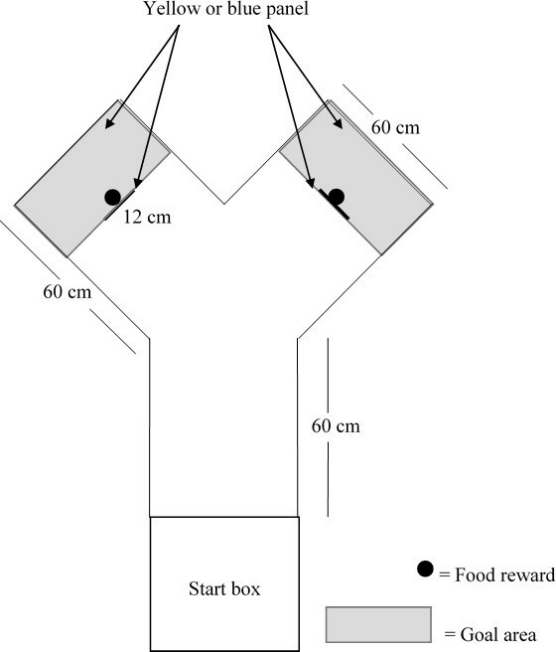
Dimensions and set-up of Y-maze and start box depicting placement of vertical colored panels, food rewards and goal area. Figure not to scale.

All behavioral experiments were conducted in a Y-maze (Figure 2) in a testing room next to the home pens, with similar temperature to that of the home pens. The testing apparatus consisted of a start box (39 x 39 cm x 52 cm), which had a removable top to allow birds to be placed in the box. The start box was separated from the Y-maze by a guillotine door that could be opened by the experimenter by sliding it up. The top of the test apparatus remained uncovered to allow video recording using a JVC Camera (JVC GC-PX100BU HD Everio) positioned on a tripod at approximately 120 cm above the ground. The end of each arm of the maze and the vertical panels (12 cm thick) mounted in the center of each arm were painted either yellow or blue (51, 52). The area behind the vertical panels represented the goal area. A feed bowl was hidden behind the vertical panel in each arm which contained the feed reward consisting of commercial diet and corn. The feed reward was present in both bowls during the discrimination tasks. However, the feed in the bowl on the non-reward side was covered with a perforated plastic screen to prevent birds from obtaining the reward and to allow for control of olfactory cues. Birds could pass the panel on the right or left side to reach the goal area and consume the reward.

### Visual Discrimination and Reversal Visual Discrimination Task

The timeline in which the birds underwent the (reversal) visual discrimination tasks is described in Figure 1. All birds were habituated to the testing apparatus before conducting the experiment over a period of 4 days. During this time, the feed was freely available in both feed bowls and on the floor to encourage birds to explore the test apparatus. Following habituation, half of the birds of each treatment were assigned to a yellow colored panel, while the other half was assigned to a blue colored panel as the reward color during the visual discrimination task (day 5–10). The feed reward was only accessible in the bowl behind the panel with their assigned reward color. Each bird was tested in one session per day, equivalent to 6 trials, until they achieved a learning criterion of at least 5 correct trials for two consecutive days. A bird had a maximum of 2 min to leave the start box and complete each trial. A trial was considered correct when the bird entered the correct arm, passed the vertical panel with at least one toe, and ate the reward in the bowl without passing the barrier of the incorrect arm first. A trial was considered incorrect when the bird passed the vertical panel of the incorrect arm with at least one toe, or did not enter any arm within 2 min of beginning the trial. There was no penalty for an incorrect trial other than the inability to access the feed reward. The visual discrimination task continued until 80% of all birds reached the learning criterion. At day 38–46, birds were given a reversal discrimination task using the same paradigm. However, the reward and stimulus were reversed for each bird (i.e., initial reward color was yellow, reversal reward color was blue and vice versa).

## Statistical Analysis

All statistical procedures were conducted using SAS V9.4 (SAS Inst. Inc., Cary, NC). The model was fitted using PROC GLIMMIX based on the mixed modeling approach for randomized experiments with repeated measures with pen as the experimental unit (53). To analyse the effect of diet treatment on body weight, feed intake, feed conversion ratio, and liver parameters, a generalized linear mixed model was employed with the fixed effect of diet group (LPER, C). Initial body weight was included as a covariate in the model for body weight and feed intake. For plasma NH4 concentration and liver enzyme activity levels, initial body weight (for initial liver enzyme activity and NH4 concentration), final body weight (for final liver enzyme activity and NH4 concentration), feed conversion ratio, average daily intake, initial NH4 concentration (for final NH4 concentration, initial ALT, initial AST, initial GGT activity levels and initial AST:ALT ratio), final NH4 concentration (for final ALT, final AST, final GGT activity levels, and AST:ALT ratio, liver hemorrhagic/color scores and liver to body weight ratio) initial AST:ALT ratio (for initial NH4 concentration and final AST:ALT ratio), final AST:ALT ratio (for final NH4 concentration, liver hemorrhagic/color score, and liver to body weight ratio), liver to body weight ratio, and number of sessions required to reach the learning criterion in the reversal task were included as covariates in the model.

To analyse the effect of diet treatment on liver scores, final body weight, final AST:ALT ratio, final NH4 concentration, final GGT activity level and number of sessions required to reach the learning criterion in the reversal task were included as covariates in the model.

A similar model was used to assess the effect of diet treatment on visual discrimination and reversal visual discrimination tasks. Average body weight at time of testing, average daily gain and feed intake were included as covariates in the model for visual discrimination task performance. For the reversal visual discrimination task, the number of sessions required to achieve the learning criterion during the visual discrimination task, average body weight at time of testing, final blood NH4 concentration, final AST:ALT ratio, final GGT activity level and liver scores were included as covariates. The data was fitted with a Poisson distribution. Due to the repeated measures taken on the same group of birds an autoregressive covariance structure of order 1 was fitted. The degrees of freedom were adjusted using the Kenward-Roger method.

To identify whether liver parameters (ALT, AST, GGT activity levels, ALT:AST ratio, NH4 concentration, liver color and hemorrhagic scores, liver to BW ratio) and learning were interrelated independent of the diet treatment, a GLM model was run for each liver parameter measured with learner/non-learner as a fixed effect. A chick was considered a learner if they were able to successfully reach the learning criterion in the reversal visual discrimination task, whereas a non-learner was unable to reach the reversal visual discrimination learning criterion. The results were considered statistically significant if *p* < 0.05. All data are presented as means ± standard errors (SE), unless otherwise indicated.

## Results

On day 36 one chick died and a post mortem examination showed 5 ml of yellow-orange clear fluid within the coelomic cavity and a mildly enlarged liver. Bilaterally, lungs were congested and oedematous.

### Body Weight and Feed Intake

The effect of reducing the protein content and increasing energy content of the diet (day 21–51) on body weight, feed intake (ADI), feed conversion ratio (FCR) and average daily gain (ADG) of female chicks is shown in Table 2. Birds did not differ in their initial body weight on day 1 (*F*_(1,14.94)_ = 0.03). Reducing the protein content by 2% and increasing energy content negatively affected the final body weight of female chicks (*F*_(1,35)_ = 7.91), and ADG was significantly lower in birds fed the LPER diet (*F*_(1,__13.76)_ =11.48). Dietary treatment did not affect ADI or FCR of female chicks.

**Table 2:** Average body weight (BW) at the beginning (1d) and end (51d) of the study, average daily gain (ADG), average daily intake (ADI), and feed conversion ratio (FCR) for each treatment group. Values presented as mean ±SEM error of the mean.

Variable	LPER	*n*	Control	*n*	Pr >F
BW (g; 1d)	42.6 ± 0.49	20	42.8 ± 0.49	20	0.8606
BW (g; 51d)	3240.3 ± 44.02	19	3451.0 ± 43.95	19	0.0045
ADG (g/day/bird)	63.9 ± 0.88	19	68.1 ± 0.87	19	0.0045
ADI (g/day/bird)	110.3 ± 1.43	19	106.6 ± 1.43	19	0.0798
FCR	1.7 ± 0.03	19	1.6 ± 0.03	19	0.0852

LPER , low protein energy rich diet. Significant between treatment groups (*p* < 0.05).

### Plasma Hepatic Markers

The effect of reducing the protein content and increasing energy on liver function of female chicks is shown in Table 3. As is evident from Table 3, both LPER and control birds had higher AST than ALT activity levels and AST activity levels increased with age. Considering all birds of the two treatment groups (LPER versus control; Table 3 regular values) plasma ALT, AST, GGT activity levels, AST:ALT ratio and NH_4_ concentration did not differ at the end of the visual discrimination task (18d) or end of the reversal visual discrimination task (46d). However, when considering only the subsample of birds (Table 3, ***italics*** values) that successfully passed the reversal visual discrimination task, final AST:ALT ratios were higher in control birds (*F*_(1,__9.756)_ = 5.49). Considering all birds of the two treatment groups (Table 3, regular values), all birds had hemorrhages on the livers (score of ≥1), an indicator of mild degree of liver damage. There were no significant differences in liver hemorrhagic scores*,* mean liver color scores, or liver to BW ratio at the end of the reversal visual discrimination task (46d). However, considering the subsample of birds that passed the reversal visual discrimination task (Table 3, ***italics*** values) birds fed the standard control diet tended to have higher hemorrhagic liver scores than birds fed the LPER diet (*F*_(1,__14.58)_ = 4.43).

**Table 3:** Average concentration of plasma ammonia (U/L) and liver enzymes (U/L) at the end of the visual discrimination experiment/start of the diet treatment (18d) and at the end of the reversal (46d) experiment, along with liver hemorrhagic and color score values, and liver to body weight ratio for birds receiving a control or low protein energy-rich (LPER) diet. Values presented as mean ±SEM error of the mean.

Variable	LPER	Control	Pr >F
ALT, 18d	***3.9 ± 0.48 (n = 16)***3.8 ± 0.41 (*n* = 18)	***2.8 ± 0.50 (n = 15)***3.0 ± 0.43 (*n* = 19)	***0.1775***0.2206
ALT, 46d	***2.3 ± 0.57 (n = 16)***2.5 ± 0.34 (*n* = 18)	***3.0 ± 0.65 (n = 16)***2.7 ± 0.38 (*n* = 17)	***0.5406***0.7261
AST, 18d	***163.4 ± 5.23 (n = 16)***159.7 ± 4.52 (*n* = 19)	***152.3 ± 5.40 (n = 15)***153.8 ± 4.66 (*n* = 18)	***0.2022***0.3969
AST, 46d	***511.4 ± 60.28 (n = 13)***570.1 ± 72.00 (*n* = 19)	***694.9 ± 67.00 (n = 13)***630.8 ± 78.94 (*n* = 16)	***0.1191***0.6081
GGT, 18d	***9.2 ± 0.58 (n = 16)***9.0 ± 0.44 (*n* = 19)	***8.4 ± 0.60 (n = 15)***8.7 ± 0.45 (*n* = 18)	***0.4339***0.6721
GGT, 46d	***10.9 ± 0.83 (n = 15)***10.9 ± 0.71 (*n* = 19)	***11.3 ± 0.90 (n = 15)*** 11.1 ± 0.77 (*n* = 16)	***0.8016***0.9120
AST:ALT, 18d	***52.5 ± 8.83 (n = 15)***59.6 ± 5.89 (*n* = 18)	***56.1 ± 8.79 (n = 15)***56.8 ± 5.85 (*n* = 18)	***0.8038***0.7622
AST:ALT, 46d	***214.8 ± 18.95 (n = 13*)**244.0 ± 19.38 (*n* = 18)	***297.35 ± 20.88 (n = 12)***270.2 ± 20.32 (*n* = 17)	***0.0417***0.4203
NH4, 18d	***2.8 ± 0.10 (n = 15)***2.8 ± 0.08 (*n* = 18)	***2.9 ± 0.10 (n = 15)***2.9 ± 0.07 (*n* = 19)	***0.7747***0.4851
NH4, 46d	***3.3 ± 0.23 (n = 14)***3.2 ± 0.21 (*n* = 19)	***3.4 ± 0.24 (n = 13)***3.4 ± 0.21 (*n* = 19)	***0.7310***0.6548
Liver Color score	***1.8 ± 0.12 (n = 14)***1.9 ± 0.12 (*n* = 19)	***1.8 ± 0.13 (n = 13)***1.8 ± 0.12 (*n* = 19)	***0.8112***0.8744
Liver hemorrhagic Score	***0.98 ± 0.08 (n = 14)***1.06 ± 0.06 (*n* = 19)	***1.29 ± 0.09 (n = 13)***1.2 ± 0.06 (*n* = 19)	***0.0531***0.0856
Liver to BW ratio	***0.01 ± 0.0007 (n = 14)***0.02 ± 0.0004 (*n* = 19)	***0.01 ± 0.0008 (n = 13)***0.02 ± 0.0004 (*n* = 19)	***0.5321***0.5078

ALT , Alanine aminotransferase; AST =Aspartate aminotransferase; GTT = Gamma glytamyl transferase; NH4 = plasma ammonia. Significant between treatment groups (*p* < 0.05). Values in *italics* refer to the number of birds that successfully passed the reversal discrimination tasks; regular values refer to all of the birds that had data for all co-variates used in the models.

## Behavioral Observations

In total, 17 out of 20 chicks that were assigned to the LPER diet treatment, and 16 out of 20 chicks that were assigned to the standard control diet treatment (dietary treatment started at day 21) were able to learn the visual discrimination task (day 5–10). The number of sessions to achieve the learning criterion for the visual discrimination task was significantly higher (*F*_(1,__8.65)_ =8.31, *p* < 0.05) in those birds assigned to the LPER (7.9 ± 0.55 sessions per bird), compared to birds assigned to the standard control birds (5.7 ± 0.47 sessions per bird). Birds assigned to LPER diet required an average of 47 trials to reach the learning criterion in the visual discrimination task, while birds assigned to the standard control diet required an average of 34 trials to reach the learning criterion (1 session = 6 trials). Three weeks after the treatment diets (LPER, control) were introduced, the number of sessions required to reach the learning criterion in the reversal task revealed no significant difference between the birds fed LPER diet (6.2 ± 0.47 sessions per birds) and those fed the standard control diet (5.5 ± 0.49 sessions per bird). Birds in both the LPER and control diet treatment groups took an average of 35 trials to reach the learning criterial for the reversal visual discrimination task.

As evident in Table 4, plasma hepatic markers, liver hemorrhage/color scores and liver to body weight were not related to achieving the learning criterion, except GGT enzyme activity, which was significantly elevated in learners (*F*_(1,__35)_ = 7.41).

**Table 4:** Average plasma ammonia (U/L) and liver enzymes (U/L) at the end of the reversal discrimination task, along with liver color and hemorrhagic scores, and liver to body weight ratio for birds that were able to learn the reversal task by reaching the learning criterion of 5 out of 6 successful sessions in two consecutive days (learners), and those that were not able to learn the reversal task (non-learners), independent of diet treatment. Mean ± SE error of means.

Variable	Learners (*n* = 28)	Non-learners (*n* = 12)	Pr >F
ALT	2.7 ± 0.25	2.4 ± 0.39	0.6000
AST	598.8 ± 47.61	644.3 ± 74.59	0.6100
GGT	11.7 ± 0.48	9.2 ± 0.76	0.0101
AST:ALT	252.3 ± 17.88	256.2 ± 28.01	0.9071
NH4	4.1 ± 0.16	4.0 ± 0.20	0.3628
Liver color score	1.8 ± 0.08	2.0 ± 0.13	0.2979
Liver hemorrhagic score	1.1 ± 0.06	1.2 ± 0.10	0.4441
Liver to BW	0.01 ± 0.0004	0.01 ± 0.0006	0.6122

ALT , Alanine aminotransferase; AST = Aspartate aminotransferase; GGT = Gamma glutamyl transferase; NH4 = plasma ammonia; Leaners = birds that were able to pass the reversal discrimination task (*n* = 19). Non-learners = birds that were unsuccessful in passing the reversal discrimination tasks (*n* = 19). Significant between learners and non-learners (*p* < 0.05).

## Discussion

This study evaluated the effects of dietary treatments, LPER and standard control diets, on, plasma hepatic markers, reversal visual discrimination performance and liver necropsy in female meat-type chickens. Birds tested in a visual discrimination task (1-10d), before going on either a LPER or a control diet, did not differ in plasma AST, ALT, GGT enzyme activity levels, AST:ALT ratio, or NH4 concentration (18d). In contrast, visual discrimination performance was inferior (slow learners) in chicks assigned to be fed the LPER diet (dietary treatment started at day 21). This indicates that the observed learning deficits in LPER birds before going on the treatment diet were not based on plasma hepatic markers, but might be dependent on individual intellectual ability, and motivation to learn a visual discrimination task (54).

The findings that LPER diets did not elevate AST, ALT, GGT enzyme activity levels, AST:ALT ratio and NH4 concentration (46d), increase liver scores (52d) or impair reversal visual discrimination learning success (38-46d) was unexpected given that previous studies indicate low protein diets increased hepatic markers (28), lead to accumulation of fat in the liver (32), and excessive intake of energy lead to reduced oxidative capacity of hepatocytes, resulting in an increased risk of developing fatty liver disease (55) in birds and produced impaired reversal learning in mammals (37, 56, 57). In the present study, we found no signs of increased liver disease in LPER birds. AST:ALT ratios, along with AST, ALT, and GGT activity levels are typically used as indicators of cell/liver damage in mammals and/or birds (26, 48, 58, 59). Unlike the present experiment, prior studies (56) used extended periods (90 days) of diet treatments and older mammals. In the present study, chicks were given unrestricted access to LPER diets but over a shorter 21 day period. This suggests that the current change in energy to protein ratio (15% increase from control to LPER diet) and duration of diet feeding in this study was not capable of eliciting the expected differences in learning and liver health.

As expected, the birds given non-restricted access to LPER diets weighed less than chicks that had access to non-restricted standard control diets. It is possible that type of diet had no significant effect on reversal visual discrimination learning partially due to weight differences and not due to the type of diet *per se*. Moreover, genetic selection for fast growth rates/high body weights in modern meat-type chickens negatively impacts walking ability(60–62). Nevertheless, chicks remain highly motivated to walk for a food reward (60). Bokkers (63) proposed that both motivation and ability to walk play a role in determining behavioral activity or inactivity in meat-type chickens. It is possible that in the present study, due to their lower body weights, birds fed the LPER diet had both higher abilities for locomotion, and higher motivation to access the luxury food reward (i.e., corn). Therefore, the ability-motivation combination may have compensated for any cognitive deficits in birds (1-10d, slow learners) fed the LPER diet (21-51d) in the present study. Similarly, Zimmermann (42) found that malnourished (fed a reduced protein diet) mammals performed equally to control mammals in a discrimination task and suggested that this could be due to the high motivation for a food reward in these animals. Additionally, a more complex learning task could elicit a stronger difference in learning abilities between the LPER and control birds, but this would need to be further examined in future studies.

In the present experiment all chicks had post-mortem signs of hepatic hemorrhage/increased liver color scores and increased AST enzyme activity levels above 230 U/L, which is indicative of hepatic damage in birds (49). In addition, LPER and control chicks showed similar GGT activity levels, where GGT values of 0–10 U/L are considered normal (46), which again suggests similar hepatic activity/damage. Interestingly, hepatic cell damage has been reported in birds dying from sudden death and in “healthy” meat-type chickens at slaughter (59). The authors concluded that liver damage in meat-type chickens may have been a result of their high feed intake, fast growth and high daily weight gain leading to high metabolic demand by the liver (59). Zuidhof et al. (64) showed that the growth rate of meat-type chickens increased by over 400% between the years of 1950 and 2005, and the average daily gain of 68 g/day observed in the control birds in the current study, further illustrate the high metabolic demand by the liver of a modern meat-type chicken. In addition, enlarged livers (paediatric hepatic lipidosis) and hepatic hematoma are among the most common medical liver problems in overweight (and hand fed) young birds (31). It is suggested that this liver cell damage/hematoma occurs because the keel provides very little protection for the underlying organs, especially in obese birds where the enlarged liver is more friable and extends further into the abdomen (31). Additionally, compromised vascular integrity can be a factor in the pathogenesis of hemorrhage in mammals/humans (65). Similar considerations could also be assumed in meat-type chickens. However, further work is needed on liver health in broiler chicks and in their parents (broiler breeders).

Birds given access to the control diet weighed more and contrary to our expectation, had significantly higher AST:ALT ratios and tended to have higher liver hemorrhage scores when passing the reversal visual discrimination task compared to LPER chicks. Enzymes such as AST, ALT, and GGT are associated with liver or muscle damage (47, 49). Kuttappan et al. (66) showed that heavier/overweight meat-type chickens were more likely to display muscle damage (damage of the sarcolemma resulting in release of various enzymes) and as a result enzyme activity levels, including AST and ALT, were increased. Despite the fact that heavier control birds had higher liver hemorrhage/damage, muscle damage in the heavier birds could be partially responsible for the increase in AST:ALT ratios in control chicks.

In conclusion, a LPER diet does not impair liver health and reversal visual discrimination learning in female meat-type chickens compared to commercial control diets. The present study suggests that due to genetic selection, the potential for growth of meat-type chickens is so high that it is overloading their liver as indicated by the high incidence of liver cell damage/liver hemorrhage, and “abnormal” AST activities in chicks regardless of whether they were fed a control or a LPER diet. This indicates that the welfare of modern meat-type genotypes is compromised and further research is needed to improve liver health in broilers.

## Ethics Statement

The experiment was approved by the Animal Care Council, University of Guelph, Guelph, ON, Canada (Animal User Protocol #3609).

## Author Contributions

LB, BP, MK and AH conceived and designed the study. LB carried out the study and analysed the data with help of NS. LB drafted the manuscript. NS, MK, and AH contributed to writing the manuscript. All authors read and approved the final manuscript.

## Conflict of Interest Statement

The authors declare that the research was conducted in the absence of any commercial or financial relationships that could be construed as a potential conflict of interest.
